# Supplementary material for: Estimating and correcting index hopping misassignments in single-cell RNA-seq data
Source: bioRxiv. 2024 Oct 24:2024.10.21.619353. Preprint. [Version 1] doi: 10.1101/2024.10.21.619353 (PMC11527012; doi:10.1101/2024.10.21.619353)
Supplement: 1 [file NIHPP2024.10.21.619353V1-supplement-1.pdf]

bioRxiv preprint doi: https://doi.org/10.1101/2024.10.21.619353; this version posted October 24, 2024. The copyright holder for this preprint (which was not certified by peer review) is the author/funder. This article is a US Government work. It is not subject to copyright under 17 USC 105 and is also made available for use under a CC0 license.

SUPPORTING INFORMATION

Figures S1-S9:

Figure S1. Skin schematic showing cells labeled by GFP in Sox2GFP/+ and Gfi1GFP/+ reporter mice.

Figure S2. Index hopping can be detected when occurring among different cell types.

Figure S3. Top DC markers genes are detected in MCs with single index hopping potential from DCs and the detection ratio is correlated with number of different cell types with the potential for index hopping.

Figure S4. Rank ordered scatter plot showing distribution of gene detection ratios in MCs across all genes from mm10 genome annotated by Ensembl.

Figure S5. Index hopping correction reduces the detection of non-self marker genes in different cell types.

Figure S6. Index hopping correction reduces the detection of non-self marker genes.

Figure S7. Assigned developmental stages of single DCs are altered by index hoping correction.

Figure S8. Assigned developmental stages of single HCs are altered by index hoping correction.

Figure S9. Detection ratios of top MC marker genes in HCs show that index hopping is reduced on Hiseq2500 compared to Hiseq3000 sequencing platforms.
